# Tardive Dysphoria: can antidepressants cause depression?

**DOI:** 10.1192/j.eurpsy.2024.1101

**Published:** 2024-08-27

**Authors:** C. P. Desport, D. O. Martins, J. R. Freitas, M. F. Santos, L. C. de Castro

**Affiliations:** Psychiatry, Hospital de Magalhães Lemos, Centro Hospitalar e Universitário do Porto, Porto, Portugal

## Abstract

**Introduction:**

tardive dysphoria is a relatively new term used to describe the phenomenon of clinical worsening of depression after long-term antidepressant use. Most of the theories proposed to explain this talk about antidepressants tachyphylaxis that implies the loss of efficacy with its prolonged use, or even a pro-depressant effect of antidepressants when used for long periods of time.

**Objectives:**

to explore the concept of tardive dysphoria, potential causes and clinical implications, by making a literature review on the topic. Moreover we pretend to understand the challenges in its diagnosis and treatment.

**Methods:**

bibliographical search in PubMed database, using the key-words “long-term antidepressant”, “tardive dysphoria” and “antidepressant tachyphylaxis”, limited to works published in the last twenty years.

**Results:**

from our search resulted 53 articles, 26 were chosen for further analysis.

**Conclusions:**

the concept of tardive dysphoria is controversial, namely doubt persists if it constitutes a clinical entity by itself caused by long-term antidepressant use or if it simply relates to cases of treatment-resistant depression. We conclude that it is necessary further investigation in this area given the significant implications on clinical practice specifically in the psychopharmacological treatment with antidepressants, which is very common in psychiatric and general practices, with antidepressants being used to treat many mental health conditions.

**Disclosure of Interest:**

None Declared

